# Immigrants’ Experiences and Perceptions of COVID-19 Information in Norway

**DOI:** 10.3390/ijerph20146421

**Published:** 2023-07-20

**Authors:** Seila Mahic, Line Nortvedt, Lise-Merete Alpers

**Affiliations:** 1 Centre of Diaconia and Professional Practice, VID Specialized University, P.O. Box 184, Vinderen, 0319 Oslo, Norway; 2 Faculty of Health Sciences, OsloMet—Oslo Metropolitan University, P.O. Box 4, St. Olavs Plass, 0130 Oslo, Norway; lino@oslomet.no; 3 Faculty of Health Studies, VID Specialized University, P.O. Box 184, Vinderen, 0319 Oslo, Norway; lise-merete.alpers@vid.no

**Keywords:** COVID-19, pandemic, immigrants, information sources, trust, qualitative, focus-group interviews

## Abstract

When the COVID-19 virus hit the world, immigrants were overrepresented among those infected. In Norway, throughout the pandemic, there were far higher infection rates in people of Somali and Pakistani descent than in the rest of the population. The first aim of this study is to explore the experiences and perceptions of immigrants from Somalia and Pakistan living in Oslo regarding the different sources of COVID-19 information that they referred to at the beginning of the pandemic. The second aim is to suggest and discuss measures to improve practice in the event of a new pandemic. Four semi-structured focus-group interviews were conducted with a total of 27 first-generation immigrants from Somalia and Pakistan. The results showed that the immigrants found various COVID-19 information sources to be important. These sources are further presented in three categories: (1) COVID-19 information from the authorities through mass communication; (2) family, friends, and local environments as sources of information; (3) information from religious communities and volunteer resource personnel. We conclude that the participants were active users of available information from different sources and channels. Stigmatization made a negative contribution and religion made a positive contribution to coping and to trust in the authorities.

## 1. Introduction

COVID-19, known more generally as the coronavirus, is an infectious disease caused by the SARS-CoV-2 virus and was first detected in China in December 2019. It quickly spread across the world, including to Europe, and thus Norway. During January–March 2020, the virus outbreak developed into an epidemic; the World Health Organization (WHO) then declared it a pandemic on 11 March 2020 [1,2]. The next day, the Norwegian government announced the most stringent measures affecting Norwegian freedoms since World War II, centered on a national lockdown, both to delay and prevent the spread of the coronavirus. Schools, nurseries, leisure activities and several workplaces were closed, a high proportion of jobs were also carried out from home offices, and social distancing was enforced. In addition, the government gave daily press conferences updating and amending their advice and regulations [3]. In the emergency plan for outbreaks of serious infectious diseases, the Ministry of Health and Care Services in Norway notes that vulnerable subgroups must be identified and that communication measures must be adapted to target groups’ needs [4]. In reality, during COVID-19, the measures were aimed at the whole population, and information was presented without specific messages for different subgroups.

As the impact of COVID-19 spread, it became clear that there was an overrepresentation of some immigrant groups, nationally and internationally, with regard to both infection and hospitalization rates, compared to the majority of the population [5,6]. At the beginning of 2023, there were 1,091,037 people with an immigrant background (persons born abroad to two foreign-born parents and four foreign-born grandparents, and persons born in Norway to two foreign-born parents and four foreign-born grandparents) living in Norway, which constitutes 19.9% of the population [7]. In Oslo, the capital of Norway, where this study was conducted, immigrants constitute 33.8% of the population [8]. Among the largest immigrant groups in Norway are people from Somalia and Pakistan [7]. These two groups saw a far higher prevalence of COVID-19 infection in their communities than the rest of the population throughout the pandemic [9]. How the infection-control measures and protocols are complied with by the population is influenced by several factors, such as the living and working situation, perceptions of risk, the degree of trust in the authorities or understanding of the messages. Compliance is also affected by the extent to which the authorities’ information reaches the population and the groups the measures are aimed at. As is well known, the population with an immigrant background is not a homogeneous group, nor do they necessarily differ from the majority population when it comes to following advice. Certain minority groups, however, had greater challenges in following the COVID-19 measures and protocols, given socioeconomic and/or structural conditions in their everyday life [10]. These conditions include cramped housing, and the fact that some immigrants were overrepresented in occupations that involve a lot of contact with others [11], such as taxi driving, cleaning, shop work, and health work. Other reasons claimed to explain why certain immigrant groups were particularly exposed to coronavirus infection include place of residence, multiple and long-term stays abroad, delayed testing, lack of infection tracing and quarantine due to language barriers, low health literacy, and conditions such as low income and low education [12].

With no curative treatment or vaccine available at the beginning of the pandemic, the WHO [13] highlighted, in addition to infection-control measures, the importance of being well informed about the disease. An Australian qualitative study shed light on how the general population in Australia learnt about COVID-19 and what sources of information they found useful and valuable early in the virus outbreak. The study showed that traditional media, online media and in-person information were important sources. It was highlighted that constantly changing news brings about challenges in terms of effective communication of risk and containment advice, and the researchers emphasized that clarity and consistency in risk messaging is important [14]. Due to the high prevalence of infection among some groups of immigrants, the need to reach out with targeted information to the immigrant population is of great importance [15].

However, one of the main challenges for public authorities in the Nordic countries was to effectively reach immigrants with key messages regarding social distancing, the need for self-isolation in case of infection and exposure, and testing and vaccination [16]. One report showed that assumptions and an individual’s life situation contribute to the challenges the authorities face in their communication work. Factors that come into play include language competence, digital skills, socioeconomic background, period of residence, transnational ties, and grounds for residence. The individual’s knowledge of public administration, connection to society, and experiences in the country of origin and the host country, as well as trust in authorities, play an important role [17]. The Nordic countries differ from other European countries when it comes to the degree of trust the population puts in the authorities [18]. A quantitative study on information access, trust, and compliance with health advice in connection with COVID-19 among immigrants in Norway showed that the majority of respondents had received sufficient information and reported high levels of trust in the Norwegian government and health authorities [19]. Some immigrants, on the other hand, had a higher level of distrust due to their experiences of unemployment, persistently low income, and discrimination and/or low participation in communal arenas [18]. In addition, different expectations due to a different cultural background may lead to distrust [20].

Although the topic of COVID-19 information has already been explored to some degree, we could not find qualitative studies that included both immigrants and their experiences and perceptions of the information they received about COVID-19. The aim of this study is, therefore, to first explore the experiences and perceptions of the different sources of COVID-19 information that immigrants from Somalia and Pakistan living in Oslo referred to at the beginning of the pandemic, and, secondly, to suggest and discuss measures to improve practice in the event of a new pandemic. 

The research question is: What kinds of sources did the immigrants refer to in connection with COVID-19 information, and how did they experience and perceive the information from those sources?

## 2. Materials and Methods

### 2.1. Study Design

This study used an exploratory and interpretive design based on hermeneutic methodology to explore the experiences of immigrants from Somalia and Pakistan of the information they received about COVID-19 at the start of the virus outbreak. An exploratory approach is relevant when knowledge of a topic is limited and new angles and information on a topic are desired [21]. At the same time, an interpretive approach is important for exploring and describing study participants’ understanding and experience more fully [22]. The Consolidated Criteria for Reporting Qualitative Research (COREQ) was followed. COREQ is a 32-item checklist that we as researchers use to report important aspects of the research team, study methods, context of the study, findings, analysis and interpretations [23].

### 2.2. Participants and Recruitment

To achieve sufficient and highly relevant information, we aimed to include participants who possessed characteristics that were specific to the study aim [24]. Inclusion criteria were, therefore, that participants were first-generation immigrants from Somalia and Pakistan of at least 18 years of age, and that they were able to choose whether they wanted to participate and were linguistically capable of being interviewed. The participants were recruited through contacting various organizations and mosques in Oslo via email and telephone. The first author (SM) and last author (LMA) also set up appointments after Friday prayers at mosques in Oslo for the purpose of recruiting participants. In total, 27 participants were recruited for this study (Table 1).

### 2.3. Focus-Group Interviews

Four focus-group interviews with the confirmed participants were conducted in groups of five to eight people. We chose focus-group interviews as this method is suitable for producing data about social groups’ interpretations of information, interactions, and norms by facilitating discussions [25]. The focus groups consisted of eight Pakistani women, seven Somali women, five Pakistani men, and seven Somali men, respectively. The division into gender was made for cultural reasons. We chose to have people in each group who were fairly homogeneous to promote a comfortable group dynamic and make it easier for the participants to express their views [26].

Data were collected from October 2021 to June 2022. Two researchers were present at all four focus-group interviews. The first author (SM) served as a discussion leader, guiding the discussions until all topics had been exhaustively covered. The last author (LMA) took notes and checked whether all topics had been covered. The interviews were carried out on premises belonging to the respective mosques or organizations, and conducted in Norwegian. A professional interpreter assisted during the interviews with the Pakistani men, but there was no need for an interpreter at the other interviews.

The first author (SM) introduced herself, the co-moderator (LMA), and the project before starting the interviews. A semi-structured interview guide was used. The guide contained questions about how the participants gained information on COVID-19 at the start of the pandemic and about their experiences with the information. Neither the transcripts nor the findings were returned to the participants for comment or correction, but the researcher often asked the participants if she had understood them correctly during the interviews. In addition, both researchers also asked follow-up questions during discussions and encouraged the participants to talk freely about the topics in the guide and to tell stories in their own words [26]. This approach is similar to hermeneutic methodology, where a new understanding of a phenomenon arises through continuous processes. A new understanding about parts of an issue influences the understanding of the issue as a whole, while new knowledge of the issue as a whole influence the understanding of the parts under observation [27]. At the conclusion of the four focus-group interviews data saturation had been achieved; the participants no longer seemed to provide new information relevant to the study topic [26]. The interviews lasted from 36 to 60 min (mean: 50.25 min).

### 2.4. Data Analysis

The first author (SM) audio recorded all the interviews and transcribed them verbatim. Field notes made during the interviews helped the researchers recognize which participant was talking at a given point. Data were then analyzed using the Graneheim and Lundman [28] content analysis method; that is to say that a “manifest content” approach was used, with a focus on the immediate, i.e., what was visible and clear in the text.

The transcribed texts were read several times to get an overview of the material. In the next step, the quotes that contained meaning units in relation to the research question were marked and color coded, and each interview was inserted into a table consisting of the following columns: meaning-bearing unit, condensed meaning-bearing unit, code, category, and theme. The quotes that were inserted as meaning-bearing units were condensed in the third stage of the analysis, and the units were divided into several categories and formed the basis for further analysis. In the fourth and final step of the analysis, abstraction involving groups of categories was put into context and assigned a common theme [28]. All four focus-group interviews offered rich data in relation to the participants’ experiences with the information provided about COVID-19. Analysis was carried out by the first author (SM) in collaboration with the other authors (LN and LMA). Three categories were identified and distributed under one theme: (1) Various sources of information and the immigrants’ perceptions of this. Table 2 shows examples from the analysis process.

### 2.5. Ethical Considerations

This study was assessed by the Norwegian Agency for Shared Services in Education and Research (SIKT), reference number 106529, which concluded that the processing of personal data was lawful and complied with data protection legislation. We complied with ethical principles for research as set out in the Declaration of Helsinki: informed consent, consequences, and confidentiality [29]. All potential participants received a short information letter from the researchers either in Norwegian or their mother tongue (Urdu or Somali), and were informed that participation was voluntary and anonymous and that at any time during the research process they were free to withdraw permission to continue the research. Most of the participants signed the informed consent form attached to the information letter, while a few with low literacy gave verbal consent to participate after receiving oral information. Confidentiality was ensured throughout this study. Interview recordings were deleted after transcription. All identifying information was removed when working with the results to ensure anonymity, and participants were assigned numbers.

## 3. Results

### 3.1. Immigrants’ Experiences and Perceptions of Various Sources of COVID-19 Information

Throughout the four interviews it was clear that the immigrants made use of various COVID-19 information sources, even though there was a lack of adapted information from the authorities at the very beginning of the pandemic. Their experiences and perceptions of these sources are further presented in three categories: (1) COVID-19 information from the authorities through mass communication; (2) family, friends, and local environments as sources of information; (3) information from religious communities and volunteer resource personnel.

### 3.2. COVID-19 Information from the Authorities through Mass Communication

For several of the participants, the main sources of the authorities’ COVID-19 information were Norwegian national TV channels and the website of one of Norway’s biggest newspapers (www.vg.no). However, according to some participants, information about COVID-19 in Norway from the authorities was only available in Norwegian during the first phase of the pandemic. One of the Somali women commented: *“Those who understand Norwegian have received enough information, but those who cannot speak or understand Norwegian, it was not easy for them”* (SW1). Other participants described how the first wave of infection affected the Somali community, in particular, and that translated written information was not available: *“During the first wave, several Somalis were infected because they either drive taxis or work in first line services. After all, they were the ones who got infected first, and there was little information available to them about what was going on and people could not understand everything”* (SM2). Both of these quotes emphasize that information was not linguistically adapted to the immigrant population at the start of the pandemic. Several of the participants said that for this reason they used foreign news channels in their native languages, but emphasized the importance of being updated on infection-control guidelines that applied to the host country. One of the Pakistani women commented: *“When we are in Norway, we follow all the Norwegian news and rules and whatnot. Because there are very different rules in Norway and Pakistan”* (PW6). This woman puts into words the perception that one must adapt to the measures where one lives. They also followed news provided by the Norwegian Institute of Public Health (NIPH) and Oslo municipality: *“We received information from Oslo municipality, and we also listened to the press conferences held by the government about the decisions they had made.”* (PM4).

Most of the participants talked about how they could trust the authorities with regard to the information presented via mass communication methods, but also in terms of how they handled the pandemic. One of the Somali men said: *“I trusted the NIPH. I live in Norway, and I have to comply with the laws and regulations here, and the authorities’ messages must of course be trusted”* (SM2). One of the Pakistani men pointed out that it was not necessary to agree with all the authorities’ advice and measures during the pandemic, but his loyalty to the community motivated him to do so. He said: *“We followed them blindly. It’s not that you have to agree with everything, but if something comes from the government, we have to follow it. We can disagree, but we must follow what the government decides”* (PM4). He trusted the government’s information regarding advice and measures in connection with the virus because *“they may be wrong, but they do not intend to deceive us.”* He explained that his faith requires him to be in solidarity with the country he lives in: *“Being Muslim, you have to follow the government in the country you live in. You have to be faithful to the country you are staying in. It is a condition of being a Muslim.”* Most of the participants said that they felt safe when they complied with the authorities’ coronavirus measures. 

However, some also experienced challenges in terms of staying updated on the information and rules: “The different measures following one after the other made it confusing… because something happens, then they downscale the readiness and then they increase it, so it was a little difficult” (SM2). This man pointed out that dealing with the constantly changing measures and information was challenging, since the changes happened so frequently. 

Early in the virus outbreak, the female participants, in particular, also lacked information about whether pharmacies and grocery stores would remain open. They were worried about whether they could get hold of face masks if pharmacies were closed, and if there was enough food for everyone in the stores. One of the women said: “I think there was little information about food and things [face masks], because people started hoarding a lot so there was really poor information about it” (PW2). This woman’s perception was that there was not enough practical information about things they needed to know for everyday life. Another woman described how TV channels and newspapers showed a shortage of various groceries in different countries: “The media were extreme when they showed people fighting over food and toilet paper, so you automatically get a little worried when you see what is happening in other countries in Europe. So, you think that it can happen in Norway too” (PW6). The response of this participant reveals how the media played an important role in people’s concern about news related to COVID-19. However, the participants claimed that they were less worried when the authorities clarified situations on the news—for example, when the Norwegian prime minister stated that there were enough groceries and that one should shop as normal.

At the same time, one of the Somali participants commented that some Somalis in Norway felt they received negative media publicity that associated them with the virus: *“You can’t stigmatize an entire group living in Norway. When you do, you create outsiderness”* (SM2). He explained that some felt labeled as villains who spread the disease, which in turn could have reduced the motivation to comply with coronavirus measures. Therefore, if there is a new pandemic or health crisis, several of the Somali participants hope for less stigmatization of selected groups: *“Somalis consist of several groups, one cannot treat everyone the same”* (SM1). Several pointed out that we must stick together in society, so that everyone works towards the same goal of *“having a normal society again. You want to get out, and you don’t want to be ‘locked inside’ It was really a shitty time”* (PW6). This woman emphasized how mainly staying at home was a strain, and that the longing for an everyday life without restrictions was huge.

### 3.3. Family, Friends, and Local Environments as Sources of Information

Almost all participants said that they received information from their school-age children at the beginning of the pandemic. One man said, *“Some receive messages from their children. We all have children, and the children receive information at school, and then the children come home and say, ‘Dad, this and that, he or she has corona. I have to get tested, the school is closed, etc.’”* (SM1). Similarly, the participants who had adult children said they received updated information about COVID-19 from them regularly. One of the informants stated: *“My children were constantly explaining the information from the news and newspapers. They say: ‘Mum, this and that are happening.’ They would call me then”* (SW7). Another participant, who had limited Norwegian language skills, said that she requested the latest information from her children if they did not offer it themselves: *“I was very curious about the rules and such, so if they didn’t give me updates, I always asked”* (PW4). One of the participants, who did not have children of her own and lived alone, pointed out that one should not take for granted that everyone has family and children nearby. She said: *“It’s hard when everything is closed, and everyone must be home alone. So, you get little information, because you don’t go anywhere or can have someone visit. This makes it difficult, and then it is good to be able to hear and watch the news to receive information”* (SW6). This woman described being isolated, with no opportunity to meet others, and was thus completely dependent on getting news from television and radio.

Friends were also important sources of information. One of the Somali women had been abroad early in the virus outbreak, and when she returned to Norway, she received a lot of information about COVID-19 from a friend: *“When I returned on 6 January 2020, a friend talked about the virus that infects others. She was watching the news constantly. I’m not very good at keeping up, I’m not interested, but she was always focusing on the news”* (SW2).

Several of the participants stated that they consulted friends who understood and spoke Norwegian better than themselves to clarify information and the changes in the measures the authorities constantly came out with. One of the participants said: *“Fortunately, I had a friend who worked in infection control, so I could call her and ask her about the current rules. She is also from Pakistan. So, she always helps me”* (PW6). Being able to get the most up-to-date information from friends by telephone was very reassuring for several of the participants. They mentioned social media such as WhatsApp and Facebook as means to keep in touch with family and friends.

Some of the participants also recounted that they knew others in their community who did not follow the information and restrictions related to COVID-19. One of the Pakistani participants said, *“In the beginning many people were not careful, and there were very large weddings here with 300–400 people, so there was a lot of spreading of the infection. While we and others have taken care not to visit each other”* (PW2). The fact that some in the Pakistani community did not comply with the measures was explained by one participant as follows: *“It must be that they don’t care”* (PW6). One of the participants also said that some community members thought that the virus was *“an invention, or that it is just a common cold. They don’t take it very seriously”* (PW2). These quotes may validate the belief that there is low health literacy among some immigrants.

### 3.4. Information from Religious Communities and Volunteer Resource Personnel

Several of the participants stated that they received information from their religious communities and the mosques, but noted that it took time for information to arrive. One of the women said, *“They are waiting for information from the authorities themselves, and then they would pass on the information”* (PW5).

These participants agreed that the information from the mosque should have come at the same time as information from the authorities, but they understood the circumstances that led to them receiving information later. The role of religious communities was particularly important as the pandemic went on, e.g., when the vaccine became available. The participants received positive feedback about the vaccine from resource personnel in the immigrant community: *“There was an older imam, more than 60 years old now. He has quit now but gives out some information if it’s important. He took the vaccine himself and said that everyone should take it and that there aren’t any very big side effects, so it’s a safe thing for us to do. So, then I got information from the mosque that it is safe to take the vaccine”* (PW2). One of the mosques had also set up a live transmission on a Norwegian website for Muslims (www.islamnett.no, accessed on 15 February 2023), where one could ask a doctor and imams questions about COVID-19 and the vaccine. One of the women said that some people wondered whether the vaccine was considered halal [i.e., anything that is considered permissible and lawful, according to Islam]: *“There were also many who wondered about haram* [*i.e., forbidden and punishable, according to Islamic law*] *and halal, so the imam sits there and says that there is no haram in medicines or vaccines, and the doctor stressed how important it was to take the vaccine*” (PW6). Several of the participants said it was reassuring for them to hear this from an imam they knew well.

Although campaigns were gradually launched by both the authorities and religious communities, the participants knew several people who did not want to get the vaccine. *“They receive the same and all the information, but they don’t want to listen. They think in their heads that corona doesn’t exist. Therefore, they don’t want [a vaccine] either”* (PW6). This participant believed that ignorance was a reason why some did not want to be vaccinated. In addition, many were afraid of side effects and influenced by rumors: *“There was so much talk about it, and that if you get the vaccine, you get this and that, like you can die after three years, and if young girls get the vaccine, they can’t have children”* (PW1). This quote emphasizes that fear also played an important part in immigrants not getting vaccinated.

Voluntary resource personnel within the immigrant communities were also of great importance in the later phase of the coronavirus pandemic. One example was the work done by a voluntary telephone helpline established by Norwegian-Somali health personnel, where one could call and receive COVID-19 information in Somali. The Somali participants were very pleased with this scheme: *“They did a terrific job”* (SM3). The helpline was particularly important to participants who did not use digital tools. The website www.NorSomNews.com (accessed on 15 February 2023), a Norwegian-Somali news platform, was also launched during the pandemic, and also became an important information channel for the Somali community. It published translated interviews with, for example, the prime minister, and provided updated information on the virus. Although the participants were satisfied with the website, they pointed to a lack of updates on the website on weekends, and that they had to be connected to the internet or have a smartphone to get updates. It was also pointed out that some Somalis are still illiterate: *“Many cannot read and write in Somali here in Norway”* (SW1). One Somali man described the important role of oral communication: *“Our society is based on oral traditions… We have our mosque here and a cafeteria, so we all talk with each other. So, if someone here gets sick today from corona, everyone knows who it is. Rumors spread. Yes, so our community is based on oral traditions, and that is very important to us”* (SM1). Some participants therefore stressed that in the event of a new pandemic, information should be provided orally and in immigrants’ native languages, and that resource personnel within the various immigrant communities should be used at an even earlier phase. One of the participants summarized it as follows: *“They [the authorities] must learn something from what has happened and be more proactive when it comes to pandemics and know which individuals/groups that can spread information quickly enough”* (SM2). This participant pointed out how important it is for the authorities to have knowledge about which resources they should use, to more quickly reach out to immigrant groups with information in the event of a new pandemic.

## 4. Discussion

In this study, we wanted to explore the experiences and perceptions of immigrants from Somalia and Pakistan living in Oslo of COVID-19 information provided at the start of the pandemic, and to suggest and discuss measures to improve practice in the event of a new pandemic.

Our findings from the semi-structured interviews show that the participants used different sources and platforms to acquire information about COVID-19 early in the virus outbreak. Several of the participants described huge curiosity about the constant presentation of new information and changes in measures. Others, however, were confused, and found it difficult to keep up to date on all the different measures presented in press conferences held in Norwegian by the authorities. This finding is in line with previous research. A study by Brønholt, Langer Primdahl [30] exploring the experiences of immigrants living in Denmark in accessing health-risk information about COVID-19 highlighted the feeling of uncertainty about the measures applied. The researchers found that many immigrants with little knowledge of the Danish language and customs were unable to keep up with the changing restrictions and guidelines and the rationale behind these [30]. In addition, another report indicated that parts of the older immigrant population may have difficulty making use of or accessing digital information about COVID-19 [31]. As our findings show, some of the participants relied on friends and family to help with information, and to explain and discuss the updated measures. Countries in Asia, Africa, and southern and eastern Europe are to a greater extent influenced by a collectivist culture, where family or those closest to you are strongly linked to and dependent on each other [32,33]. The opportunity to discuss different situations with family and friends, as the participants did, is an important resourse and refers to a unique collective information-processing method, whereby participants share information they are questioning with people they trust, and then reach a conclusion together [34]. Collectivistic societies are characterized by values such as loyalty and perseverance [35]. It is therefore important that the authorities consider how they can leverage collectivist values when providing information, and also when encouraging people to continue to follow restrictions.

However, one of the participants in our study pointed out that one cannot take for granted that everyone has acquaintances they can discuss COVID-19 information with. Some are therefore dependent on receiving comprehensible information via the mass media. As a previous study also highlighted, without prevailing and understandable mass media, some people will have limited access to objective sources of information [36]. The results from the Somali group in our study show that Somali volunteers came together to create the website www.NorSomNews.com (accessed on 15 February 2023) to, among other things, translate written information that had been put out by the authorities. For several participants this was valuable, but others highlighted that illiteracy was still widespread among the Somali people and stated that information given orally is of great importance. Similar results were found by Finell, Tiilikainen [37], who described how their Somali-speaking respondents were more comfortable relying on informal oral communication when seeking information about their country of settlement. These results make sense given the strong oral culture in Somali society [38]. This may also explain why Somali health personnel in Norway saw the need to set up the helpline. Nevertheless, both the website and helpline were highly valued by our participants because of the opportunities they provided to gain information in their own language. However, the main responsibility for providing information and implementing infection-control measures lies with public authorities [31]. We therefore highlight the importance of authorities being familiar with how at-risk groups prefer to acquire information. This is important because resource personnel from immigrant communities may have limitations related to infection-control competence, continuity, and capacity over time [31]. This is in line with the findings of this study, where the participants told us how there was no updated information on NorSomNews.com at weekends, and that some of them had to wait for information from the mosques.

Unavailable or missing information from professionals and authorities may also contribute to the spread of misinformation. Our results show that some participants were worried about whether there was enough food and face masks for everyone, due to a lack of information about this from the authorities and media reports about hoarding in other countries. As Liu, Hongzhong [39] pointed out in their study, media narratives play an important role in public responses to COVID-19. It is therefore recommended that government and media professionals deliver balanced information about pandemics instead of overemphasizing negative information, since this may fuel panic and uncertainty among the public.

Zou and Tang [34] highlighted in their study that rumors are a natural by-product of any crisis. Yet, they claim that the COVID-19 pandemic was different from previous infectious-disease outbreaks, such as the SARS, MERS, or Ebola outbreaks, because it was characterized by a flood of rumors, misinformation, and conspiracy theories. The participants in our study demonstrated awareness that misinformation was rife in their environments, both when it came to the disease itself and the vaccine, when it arrived. Skepticism that the virus was an invention and fear of serious side effects or death as a result of the vaccine were the most widespread rumors they had heard. Several religious organizations started working on adapted COVID-19 information to their members to cover the gap of adapted information. With time, the Norwegian authorities also began to cooperate with religious organisations to improve communication and information aimed at the immigrant population during the pandemic [3]. Our study reveals for example the role of religious communities in providing the strength to cope with the rumors, misinformation, and insecurity. The participants in our study referred to religious communities that took responsibility for their members and which arranged digital meetings where the public could ask questions both of imams and doctors. To hear from an imam they trusted and knew well that there was no haram in taking the vaccine was quite reassuring for several of the participants. This can be seen in terms of the idea that it is often easier to trust someone who is similar to oneself in terms of culture and religion [40]. In addition, several of the participants expressed trust in the Norwegian authorities and their handling of the pandemic, and said religion was a factor that promoted this trust as it provided the basis for loyalty to the country they live in. Religion is, for many immigrants, a far more important part of life than it is for the rest of the population, and those immigrants who are religiously active have somewhat stronger trust in other people than those who are less religiously active [41]. 

Based on our results, we therefore suggest that religious resource personnel should be used as early as possible in a crisis, and that the cooperation between them and the authorities be maintained even when pandemics or other crises are absent.

The Somali group of men stood out on the topic of trust. They reported feeling stigmatized as an immigrant group who spread the virus in Norwegian society. Additionally, they felt that Somali people were associated with several conspiracy theories related to COVID-19 that were reported in the media. As our results show, some of the participants claimed that such stigmatization, for some, can lead to less involvement in society and less motivation to comply with measures presented by the authorities. The experience of being treated differently, as in the case of these Somali participants—of being “labeled” because of one’s background—can also have a major effect on trust, both in other people and in the authorities [41]. It is a well-known fact that crisis tends to put trust in society to the test [42]. As Grimen [40] highlighted, it is easier to go from trust to mistrust than vice versa. Mistrust is easy to create, but difficult to get rid of, and, most importantly, it destroys opportunities for interaction. To avoid such stigmatization and the resulting threat to trust in the authorities, the alternative may be to only focus on environments that are affected—in this case, environments that had a high prevalence of infection. A similar approach was taken with regard to the mpox outbreak, where several cases were reported among gay, bisexual and other men who have sex with men. In connection with that, the Joint United Nations Programme on HIV/AIDS (UNAIDS) urged media outlets, governments, and communities to respond with a rights-based, evidence-based approach that avoids stigmatization of LGTBI people. The director of the programme, Matthew Kavanagh, has said: “Stigma and blame undermine trust and capacity to respond effectively during outbreaks like this one” [43]. One can therefore claim that effective handling of a pandemic depends on the population having trust in the authorities [44], as this can increase public compliance with official recommendations, minimize health risks, and help authorities manage the crisis [45]. After all, the COVID-19 pandemic has shown that fighting an outbreak is a joint effort: everyone must contribute if the fight is to be successful.

### Strengths and Limitations

A key strength of this study is that we managed to include people from the immigrant groups that had the highest incidence of infection in Norway. Another strength is that we had the opportunity to use an interpreter for all of the group interviews. This meant that we could include participants who did not speak fluent Norwegian or who were not confident speaking in Norwegian. In addition, it is a strength that we had two researchers who were present at all four focus-group discussions, and who discussed the findings among the three researchers. However, this study has some limitations. First, we recruited participants through voluntary organizations and religious networks. This may mean that we recruited immigrants who were more engaged in the society. Thus, we have missed out on hearing about the experiences of immigrants who were the most vulnerable. A second study limitation may be the number of participants, i.e., that number was quite small. However, the aim of qualitative studies is not to generalize, but to provide sufficient descriptive data in the form of strong descriptions so that consumers can evaluate the relevance of the data to other contexts [26]. To increase the transferability of this study, we have therefore documented the analysis process, presented the data with illustrative quotes, and described the sample closely.

## 5. Conclusions

Despite the fact that there was a lack of adapted information for immigrants at the start of the pandemic, our findings highlight that the participants in this study were active users of information from different sources and channels; it was not the case that they did not keep up to date at all. The authorities and public health authorities need to implement policies that include guidelines on how to quickly reach, and how to adapt communication and information to linguistic minorities, for example by translating all brochure material in the five largest language groups in Norway. In addition, establish collaboration partners in religious communities and other recourse persons with the various immigrant communities. There should also be clarified areas for responsibility when different actors cooperate in providing information. This can ensure that adapted information is presented from the start in case of a new pandemic. With that said, immigrants are not a homogeneous group in relation to their information needs, and in experiences during a pandemic. It is therefore important that several aspects are taken into account such as social, economic and cultural belonging. Our results reveal for example that religion was seen as important for our participants during the pandemic, both in terms of coping with misinformation regarding COVID-19 and in that it helped them put trust in the authorities during the pandemic. We suggest that a further study should be performed to investigate the experiences of religious resource personnel about their role and work during the COVID-19 pandemic. Based on our results, we highlight that stigmatization of certain groups can lead to less motivation to comply with measures presented by the authorities, and that it can affect trust in both other people and the authorities. The authorities being aware of this and addressing the topic in a more tailored way may increase public trust in them and thus help them to better navigate future pandemics and crises. We summarize the mentioned key recommendations in this study with bullet points:

- Consider how to leverage collectivist values when providing information,
- Use prevailing and understandable mass media,
- Being familiar with how at-risk groups prefer to acquire information,
- Establish collaboration partners in religious communities and other recourse persons and clarify areas for responsibility in providing information, and
- Avoid stigmatization of certain groups in the society.

## Figures and Tables

**Table 1:** Participant characteristics (*n* = 27).

Gender	
Female	15
Male	12
Age group	
31–40	2
41–50	9
51–60	6
61–70	5
71–80	5
Country of origin	
Somalia	14
Pakistan	13
Number of years lived in Norway	
10–20	16
21–30	3
31–40	4
41–50	3
51–60	1

**Table 2:** Example of the analysis process.

Meaning-Bearing Unit	Condensed Meaning-Bearing Unit	Code	Category	Theme
“We received information from Oslo municipality, and we also listened to the press conferences held by the government about the decisions they had made. So, we followed what Oslo municipality or the government had said” (PM4).	Received information from Oslo municipality and followed the press conferences held by the government about the decisions they had made.	Information from Oslo municipality and from press conferences conducted by the government.	COVID-19 information from the authorities through mass communication.	Immigrants’ experiences and perceptions of various sources of COVID-19 information.
“Those who have children at school get information from there, and there is often information there” (SW2).	When you have children in school, you often get information from there.	Information through children at school.	Family and friends as sources of information.	Immigrants’ experiences and perceptions of various sources of COVID-19 information.
“We received positive feedback from the mosque. We received information that it [the vaccine] is not dangerous and that we should take it, and that we must follow the rules. So, I read on the mosque’s Facebook page and islamnet.no as well. They had a live broadcast, and you could write down questions for them. I think it was to a doctor and some imams” (PW6).	Received positive feedback from the mosque regarding the vaccine, and were encouraged to take it. Got information from the mosque’s website and www.islamnet.no, accessed on 15 February 2023. The mosque set up a live meeting with a doctor and imams for questions.	Positive feedback from resource personnel from the mosque regarding the vaccine.	Information from religious communities and volunteer resource personnel.	Immigrants’ experiences and perceptions of various sources of COVID-19 information.

## Data Availability

Data generated during the present study cannot be shared due to the need to preserve the informants’ privacy and confidentiality.
